# Phenotypic analysis of ataxia in spinocerebellar ataxia type 6 mice using DeepLabCut

**DOI:** 10.1038/s41598-024-59187-0

**Published:** 2024-04-13

**Authors:** Dennis Piotrowski, Erik K. H. Clemensson, Huu Phuc Nguyen, Melanie D. Mark

**Affiliations:** 1 Behavioral Neuroscience, Faculty for Biology and Biotechnology, Ruhr-University Bochum, ND7/32, Universitätsstr. 150, 44780 Bochum, Germany; 2 Department of Human Genetics, Medical Faculty, Ruhr-University Bochum, Bochum, Germany

**Keywords:** Spinocerebellar ataxia type 6, DeepLabCut, Markerless pose estimation, Open-source, Gait analysis, Computational biology and bioinformatics, Neuroscience

## Abstract

This study emphasizes the benefits of open-source software such as DeepLabCut (DLC) and R to automate, customize and enhance data analysis of motor behavior. We recorded 2 different spinocerebellar ataxia type 6 mouse models while performing the classic beamwalk test, tracked multiple body parts using the markerless pose-estimation software DLC and analyzed the tracked data using self-written scripts in the programming language R. The beamwalk analysis script (BAS) counts and classifies minor and major hindpaw slips with an 83% accuracy compared to manual scoring. Nose, belly and tail positions relative to the beam, as well as the angle at the tail base relative to the nose and tail tip were determined to characterize motor deficits in greater detail. Our results found distinct ataxic abnormalities such as an increase in major left hindpaw slips and a lower belly and tail position in both SCA6 ataxic mouse models compared to control mice at 18 months of age. Furthermore, a more detailed analysis of various body parts relative to the beam revealed an overall lower body position in the SCA6^84Q^ compared to the CT-longQ27^PC^ mouse line at 18 months of age, indicating a more severe ataxic deficit in the SCA6^84Q^ group.

## Introduction

Many classical experiments testing the motoric performance of mice have been established over the last centuries. Traditionally, these tests have been scored manually by the researchers performing them. Although some of these tests, like measuring the stride lengths and widths using paint, paper and a ruler^1,2^, have become more sophisticated and automated through for example the CatWalk, LocoMouse and DigiGait^3–7^ or a self-made, simple gait analysis system using an open-field setup^8^. The automation of mouse movements during gait has provided a deeper understanding on the coordination of movement across multiple body parts and identification of abnormal, gait ataxia in a quantitative, unbiased manner under normal, unrestrained conditions. The DigiGait system even allows the researcher to challenge the subject on a treadmill with different levels of inclines and speeds for more subtle ataxic phenotypes^7^. However, subtle differences between ataxic mouse lines are sometimes not evident from normal gait analyses systems like the CatWalk and LocoMouse, unless placed under more technically challenging conditions to bring out mild ataxic differences such as the beamwalk. The beamwalk analysis can also be supportive and a verification of the gait analysis results. Many of these supportive motor coordination tests are still scored manually. This process is not only time consuming, but also prone to error and bias. In the classic beamwalk test the number of slips of any given paw will be scored, but like behavior itself, how one classifies the displacement of a paw as a slip is not well defined^9^. This classification becomes even more difficult when including phenotypes presenting distinct crossing strategies that do not fit the initial description of a slip. Furthermore, analyzing phenotypes only using discrete values, such as the number of slips, misses out on a more detailed analysis using continuous measurements like the relative position of body parts to the top of the beam. Enriching and unbiasing the analysis of this classic beamwalk test is likely to provide similar benefits like the transition from the classical foot printing test with painted paws to the CatWalk.

Many laboratories use mouse models for understanding the mechanisms underlying neurodegenerative diseases to advance the diagnostic and therapeutic possibilities for individual patients. Open-source software such as DeepLabCut (DLC) can provide a reliable, unbiased phenotypic readout of neurodegenerative disease progression in for example ataxic patients. Past studies have used open-source software such as DLC^10,11^ for markerless tracking of body parts to advance their research and to improve their analysis of characteristic motor impairments like ataxia in mice and men^12–15^. For example, a recent study applied DLC to identify tremors and abnormal ataxic behaviors in an ataxic and tremor rodent model called the shaker and a mouse model for Spinocerebellar ataxia type 3 (SCA3)^12^. Additionally, another group used the machine learning-based system JAABA (Janelia Atomatic Animal Behavior Annotator) to characterize the ataxic phenotype in a Purkinje cell-specific knockout of calcium/calmodulin-activated protein-phosphatase-2B (PP2B) mouse model for ataxia^16^. A more recent study in Parkinson’s patients utilized DLC and algorithms to objectively correlate neural signals with movement to ideally place deep brain stimulation electrodes^13^.

This study aimed to contribute with self-written scripts, written in the programming language R, to enhance the analysis of the classic beamwalk experiment using DLCs tracking in mouse models for Spinocerebellar Ataxia Type 6 (SCA6). Spinocerebellar Ataxia Type 6 is caused by an expanded polyglutamine (polyQ) repeat at the carboxy terminus (CT) of the P/Q-type calcium channel α1A subunit (Cacna1a) which leads to Purkinje cell (PC) degeneration and progressive ataxia^17,18^. Unaffected individuals normally have a polyQ expansion ranging from Q4-Q18, however SCA6 patients have a range of Q21-Q33. In humans, an alternative splicing event occurs resulting in 2 isoforms, one lacking (short) or one containing the CT polyQ expansion (long)^19,20^. Both isoform transcripts are equally abundant in adult cerebellar PC. However in individuals suffering from SCA6, the diseased long isoform transcript is doubled^19^ compared to the long isoform transcript from unaffected individuals. Moreover, the CT of the Cacna1a undergoes proteolytic degradation leading to a more stable diseased CT peptide fragment, which specifically accumulates in cytosolic and to a lesser extent in nuclear PC protein aggregates from SCA6 patients. To imitate the human SCA6 pathology, our lab demonstrated that the overexpression of 27 polyQs in the CT was sufficient to cause SCA6 like symptoms in mice such as late onset, progressive ataxia (≥ 8 months of age), PC degeneration and deficits in associative motor learning^18^. Additionally, Watase et al.^21^ generated a SCA6 knockin mouse model containing a hyperexpanded 84 polyQ tract in the CACNA1A locus which also showed late onset, progressive ataxia and aggregation of the hyperexpanded 84 polyQ tract. Interestingly the length of the polyQ repeats is inversely correlated to the age of onset and directly correlated to the severity of the disease in humans.

In order to characterize the disease progression between these 2 different genetically modified mouse models for SCA6^18,22^ which were not evident in classical gait analyses and manually scored supportive motor coordination tests including the beamwalk, pole test and hangwire, we first validated the automated scoring of the self-written script which we coined as beamwalk analysis script (BAS) by comparing the BAS to manual scoring performed by a trained researcher. Once the BAS was validated, various body parts such as left forepaw, left hindpaw, nose, belly, back and tail positions were determined at preonset and late onset SCA6 disease stages for evaluation of their ataxic phenotypes.

## Results

### Validation of BAS

To initially evaluate the accuracy and validity of the trained DLC model as well as the slip classification of the self-written R script, 2 different SCA6 mouse models were compared to control mice. One was a mouse line with a CRE-regulated transgenic overexpression of CT containing an expanded polyQ repeat of 27 glutamines (similar amount found in SCA6 patients). The overexpression was specifically targeted to cerebellar Purkinje cells (PCs) postnatally by crossing them with the transgenic Pcp2-CRE mice where CRE recombinase is under the control of a PC specific promoter^18^. These mice were referred to as CT-longQ27^PC^ and were compared to their littermate transgenic Pcp2-CRE only or CT-short^PC^ controls, where only the CT without a polyQ domain was overexpressed in PC postnatally. The second SCA6 mouse model characterized contained a knockin of 84 polyQ repeats into the α1A subunit of the P/Q-type calcium channel^21^. Both SCA6 mouse lines display progressive ataxia after 7 months of age, PCs degeneration and altered PC firing. To investigate if the markerless motion tracking could detect subtle changes in ataxic behavior, we tested SCA6 mice at preonset disease stage of 6 months and late onset disease stage of 18 months.

We first confirmed that there were no large differences in body size between the various mouse lines which would need to be considered in our analyses. The average area between the marked points for the nose, belly, tail base and back were calculated for every frame as an irregular rectangle for each mouse. There were no differences in the mean size (11.54 ± 0.1 cm^2^) for all mice at 18 months of age (Supplementary Table 1). However, SCA6^84Q^ mice at 6 months were smaller than controls and CT-longQ27^PC^ mice (p = 0.0029) without affecting the phenotype. Therefore, we did not consider body size differences in our calculations.

**Figure 1:**
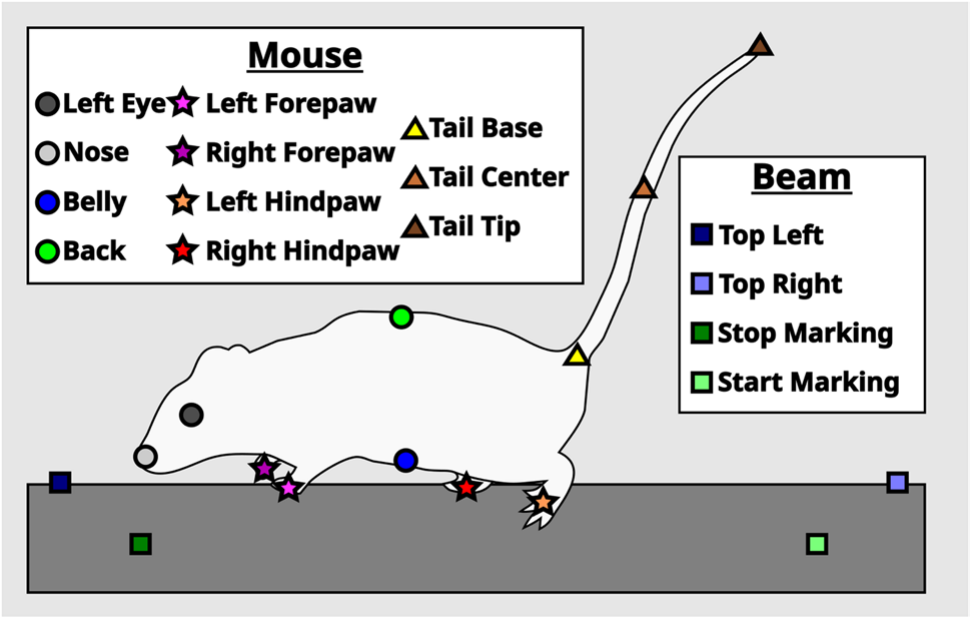
Schematic of all body parts and beam positions tracked by DeepLabCut. Stars represent points tracked on paws where the left forepaw (pink star) and the left hindpaw (orange star) were used in the analysis. Similarly, triangles represent points tracked on the tail which includes the tail base (yellow triangle), the tail center (light brown triangle) and the tail tip (dark brown triangle). Circles represent all other body parts that were tracked. This includes the nose (light grey circle), the back (green circle) and the belly (blue circle). The left eye (dark grey circle), right forepaw (purple star) and right hindpaw (red star) were not used in the analysis and were only tracked for completion. Squares represent points tracked on the beam. Two points tracked the top of the beam on the left (dark blue square) and the right (light blue square) side. Two markings placed on the beam indicated the end (dark green square) and the start (light green square) of the trial during the experiment and analysis.

**Figure 2:**
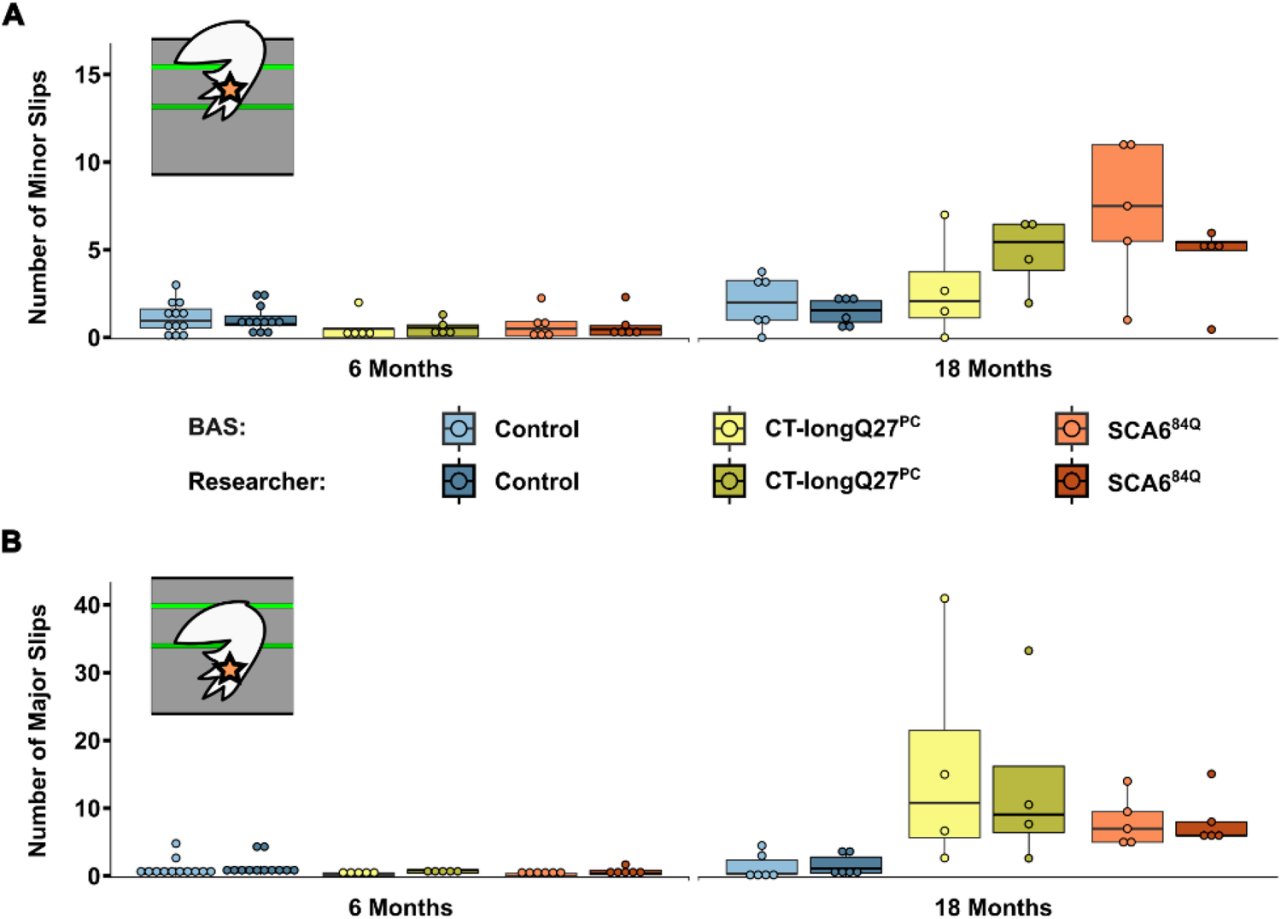
Performance comparison between the beamwalk analysis script (BAS) and researcher. The BAS utilizes the coordinates retrieved from DeepLabCut (DLC) to classify vertical left hindpaw (orange star) displacements as minor and major slips. For this analysis, minor slips were defined as paw positions between 20% (upper green line) and > 50% (lower dark green line) below the beam. Any slips > 50% (lower dark green line) below the beam were defined as major slips. A trained researcher analyzed the data frame by frame using the same rules as the BAS. Two different SCA6 mouse models (CT-longQ27^PC^ and SCA6^84Q^) at different disease stages (6 and 18 months), compared to control mice were used. (**A**) Comparison of counted minor slips by BAS (lighter shades) and a trained researcher (darker shades). (**B**) Comparison of counted major slips by BAS (lighter shades) and a trained researcher (darker shades). No significant differences between the BAS and researcher were found. Statistical significance was evaluated using two-tailed Mann–Whitney *U*-tests. Shown are boxplots of the mean number of slips over all trials of each mouse represented as individual dots.

Next, we confirmed the accuracy of the DLC tracking and BAS by comparing the number of minor and major left hindpaw slips (marked as an orange star in Figs. 1 and 2) counted by BAS and a trained researcher who analyzed the data frame-by-frame using the same thresholds. Minor slips were defined as hindpaw positions between 20 and 50% below the top edge of the beam, and major hindpaw slips as > 50% below the top edge of the beam (Supplementary Video 1). No significant difference in scores between the researcher and BAS were identified (Fig. 2). Ninety trials were counted by BAS for minor slips with a ± 1 difference to the researcher and with an ≈78% accuracy. For scoring major slips, 102 trials were counted by BAS with a ± 1 difference to the researcher and with an ≈89% accuracy. Overall minor and major slips were counted by BAS displaying an ≈83% accuracy compared to the researcher, indicating that the DLC tracking and BAS were both functioning with a satisfactory accuracy.

### Classification of major and minor paw slips and quantification of relative paw position on the beam reveals ataxic motor impairments

**Figure 3:**
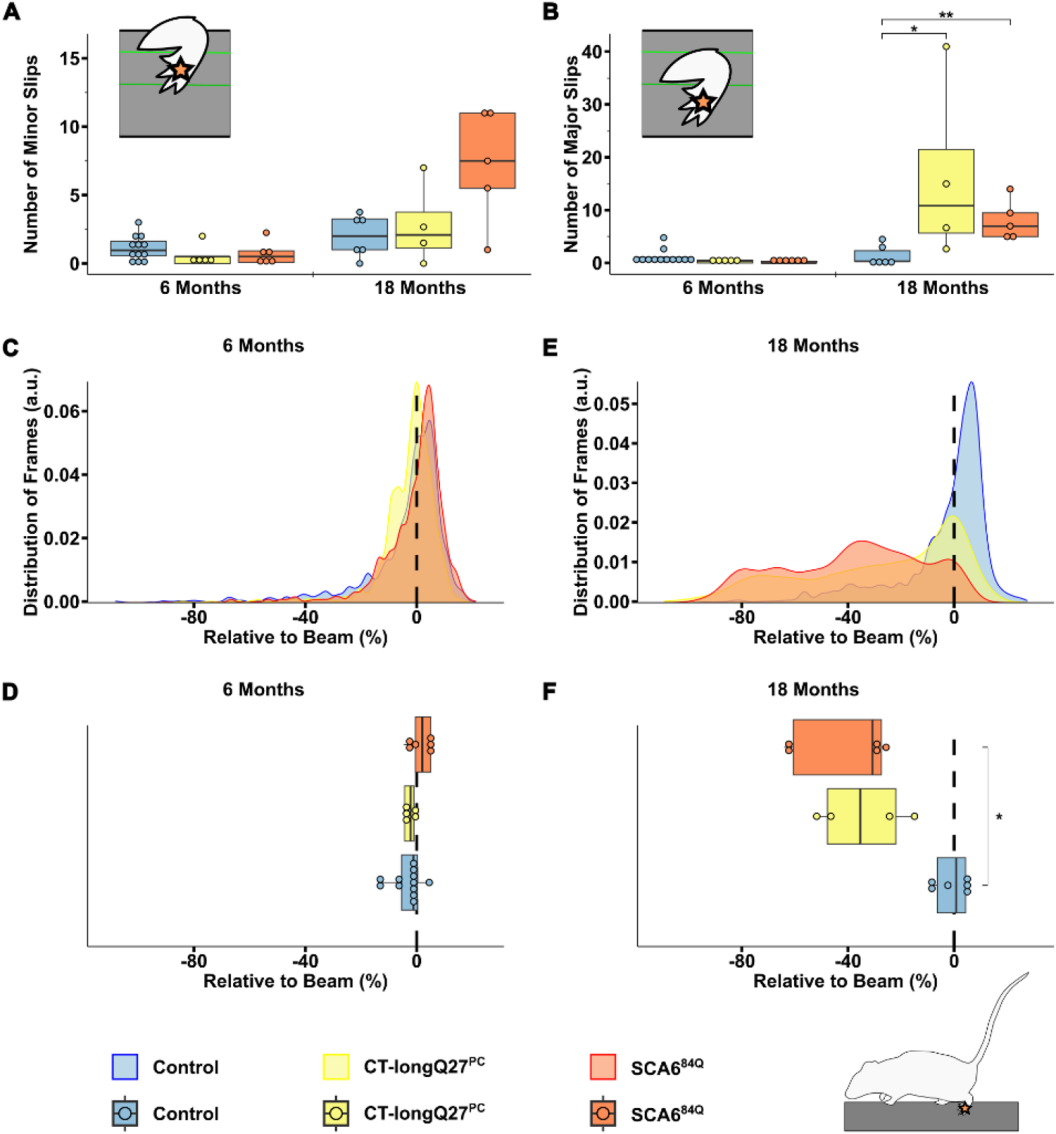
Classical and advanced analysis of the left hindpaw by the beamwalk analysis script (BAS). The BAS utilizes the coordinates retrieved from DeepLabCut (DLC) to classify vertical left hindpaw (depicted in scheme as orange star) displacements as minor (**A**) and major (**B**) slips. For this analysis, minor slips were defined as paw positions between 20% (upper green line) and 50% (lower dark green line) below beam. Any slips below 50% were defined as major slips. The left hindpaw position relative to the top of the beam in percent was analyzed in (**C**–**F**), where a vertical dashed line represents 0% (the top of the beam) on the x-axis. Two different SCA6 mouse models (CT-longQ27^PC^ and SCA6^84Q^) at different disease stages (6 and 18 months), as well as control mice were compared to each other. (**A**) Boxplots of the mean number of minor slips over all trials. No significant difference was found between groups at 6 months of age. (**B**) Boxplots of the mean number of major slips over all trials at 18 months of age. At 18 months of age control mice significantly slipped less compared to CT-longQ27^PC^ (p = 0.04) and SCA^84Q^ mice (p = 0.0074). (**C**) Density plot displaying the probability distribution of the left hindpaw position relative to the top of the beam from 6 months old mice. (**D**) Boxplots of the mean left hindpaw position relative to the top of the beam from 6 months old mice. No significant difference was found between groups at 6 months of age. (**E**) Density plot displaying the probability distribution of the left hindpaw position relative to the top of the beam from 18 months old mice. (**F**) Boxplots the mean left hindpaw position relative to the top of the beam from 18 months old mice. The left hindpaw of SCA^84Q^ mice were placed lower on the beam compared to control mice (p = 0.014). Statistical significance was evaluated using two-tailed Mann–Whitney *U*-tests in (**A**), (**B**), (**D**) and (**F**). Bonferroni-Holm was used to correct for multiple testing. Each tested mouse is represented as individual dot. **p < 0.01; *p < 0.05.

To determine if there were detectable differences in minor and major paw slips between the SCA6 mouse lines compared to controls, we applied the BAS and found no changes in the number of minor slips (Fig. 3A) or position of the left hindpaw relative to the top of the beam (Fig. 3C and D) between mouse lines at 6 months of age. However, large discrepancies in major, left hindpaw slips were demonstrated at 18 months in both CT-longQ27^PC^ (16.33 ± 8.61, p = 0.04) and SCA^84Q^ (8.10 ± 1.69, p = 0.0074) mice compared to controls (1.42 ± 0.76, Fig. 3B). Additionally, SCA6^84Q^ mice (− 42.37 ± 10.35, p = 0.014) were likely to move their left hindpaw below the beam compared to control (− 1.73 ± 3.27, Fig. 3F) mice at 18 months of age. Although 18 months old CT-longQ27^PC^ mice (− 32.28 ± 10.75, p = 0.06, Fig. 3F) did not show any significantly different paw positioning compared to control mice, the density plots (Fig. 3E) depicted a left shift in left hindpaw placement relative to the beam which was more evident in the SCA^84Q^ mice. These results indicate that both SCA6 mouse lines tended to place their left hindpaw below the edge of the beam and that SCA^84Q^ mice show a more severe ataxic phenotype than CT-longQ27^PC^ mice. Unexpectedly, there were no differences in minor and major left forepaw slips in all mouse lines at both ages, even with lower thresholds for minor and major slips (Supplementary Fig. 1A and B). Six month old SCA6^84Q^ mice positioned their left forepaw slightly higher on the beam than CT-longQ27^PC^ and control mice (Supplementary Fig. 1C and D). At 18 months of age CT-longQ27^PC^ mice placed their left forepaw lower on the beam than controls (Supplementary Fig. 1E and F).

### Classification of nose, belly and tail position

**Figure 4:**
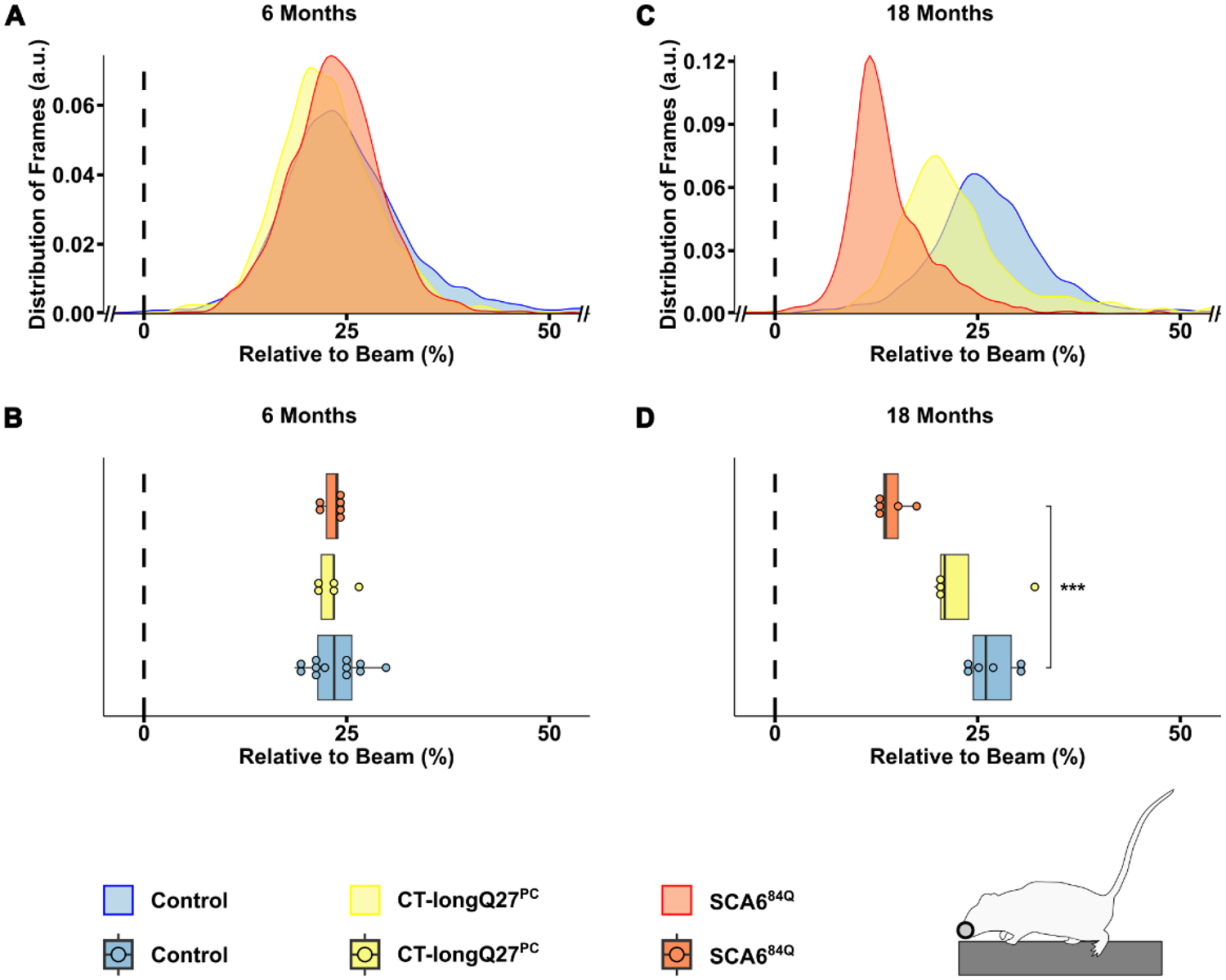
Analysis of nose position relative to the beam by the beamwalk analysis script (BAS). The BAS utilizes the coordinates retrieved from DeepLabCut (DLC). (**A**) Density plot of nose position relative (depicted in scheme as grey circle) to the top of the beam from 6 months old mice. The relative nose position to the top of the beam in percent. The top of the beam is represented as a vertical dashed line as 0% on the x-axis. Two different SCA6 mouse models (CT-longQ27^PC^ and SCA6^84Q^) at different disease stages (6 and 18 months), as well as control mice were compared to each other. For better visualization the data is zoomed in on the x-axis in (**A**) and (**C**). (**B**) Boxplots of the mean relative nose position to the top of the beam from 6 months old mice. No significant difference was found between groups at 6 months of age. (**C**) Density plot of nose position relative to the beam from 18 months old mice. (**D**) Boxplots of the mean relative nose position to the top of the beam from 18 months old mice. SCA^84Q^ mice were more likely to place their nose closer to the beam compared to control mice (p = 0.000095). Statistical significance was evaluated using two-tailed Mann–Whitney *U*-tests in (**B**) and (**D**). Bonferroni-Holm was used to correct for multiple testing. Each tested mouse was represented as individual dot. ***p < 0.001.

**Figure 5:**
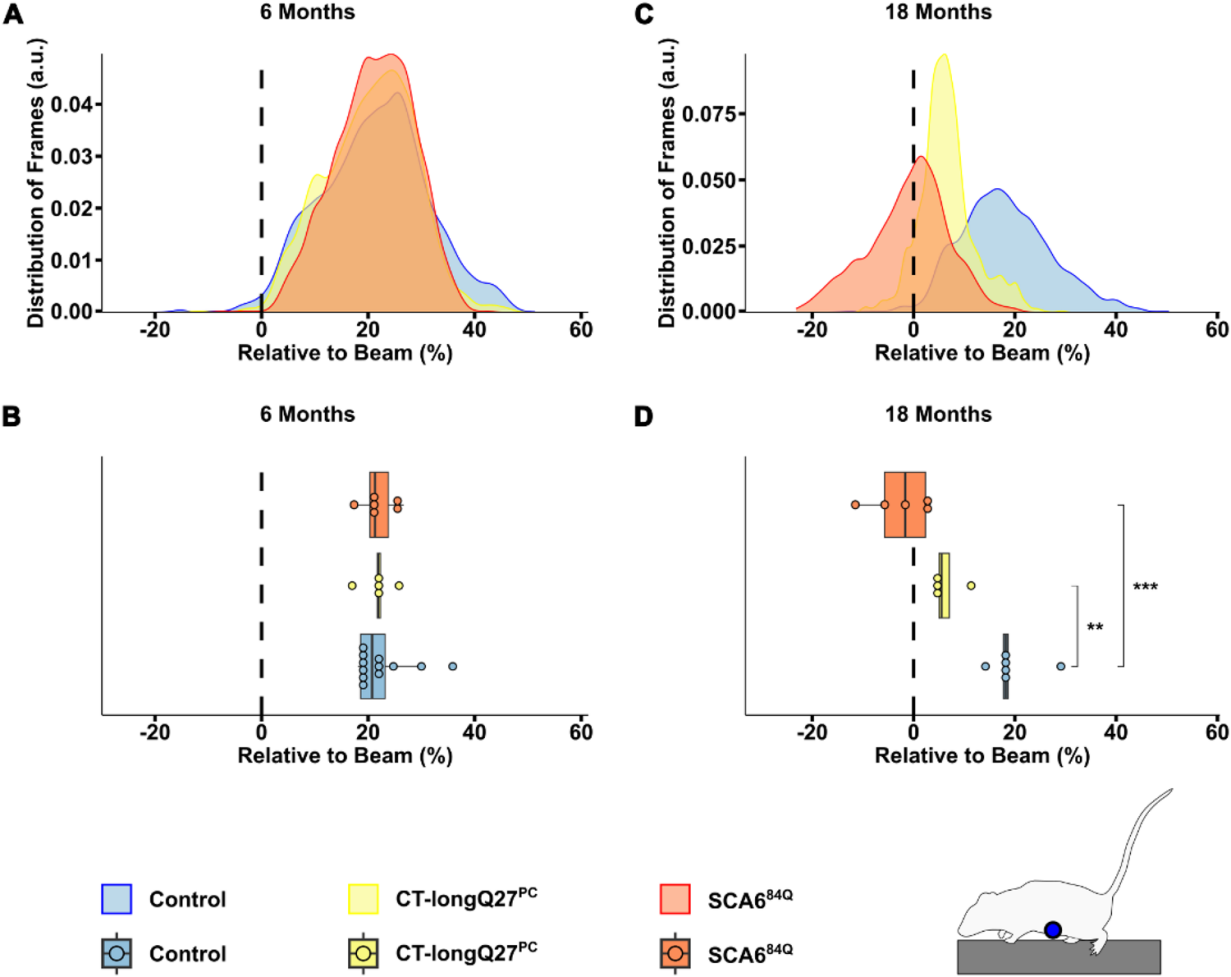
Analysis of belly position relative to the beam by the beamwalk analysis script (BAS). The BAS utilizes the coordinates retrieved from DeepLabCut (DLC). (**A**) Density plot of belly position (depicted in scheme as blue circle) relative to the top of the beam from 6 months old mice. The relative belly position to the top of the beam in percent, where the top of the beam is represented as a vertical dashed line at 0% on the x-axis. Two different SCA6 mouse models (CT-longQ27^PC^ and SCA6^84Q^) at different disease stages (6 and 18 months), as well as control mice were compared to each other. (**B**) Boxplots of mean relative belly position to the top of the beam from 6 months old mice. No significant difference was found between groups at 6 months of age. (**C**) Density plot of belly position relative to the top of the beam from 18 months old mice. (**D**) Boxplots of mean relative belly position to the top of the beam from 18 months old mice. CT-longQ27^PC^ (p = 0.0021) and SCA^84Q^ mice (p = 0.0005) were more likely to place their belly closer to the beam compared to control mice. Statistical significance was evaluated using two-tailed Mann–Whitney *U*-tests in (**B**) and (**D**). Bonferroni-Holm was used to correct for multiple testing. Each tested mouse is represented as individual dot. ***p < 0.001; **p < 0.01.

**Figure 6:**
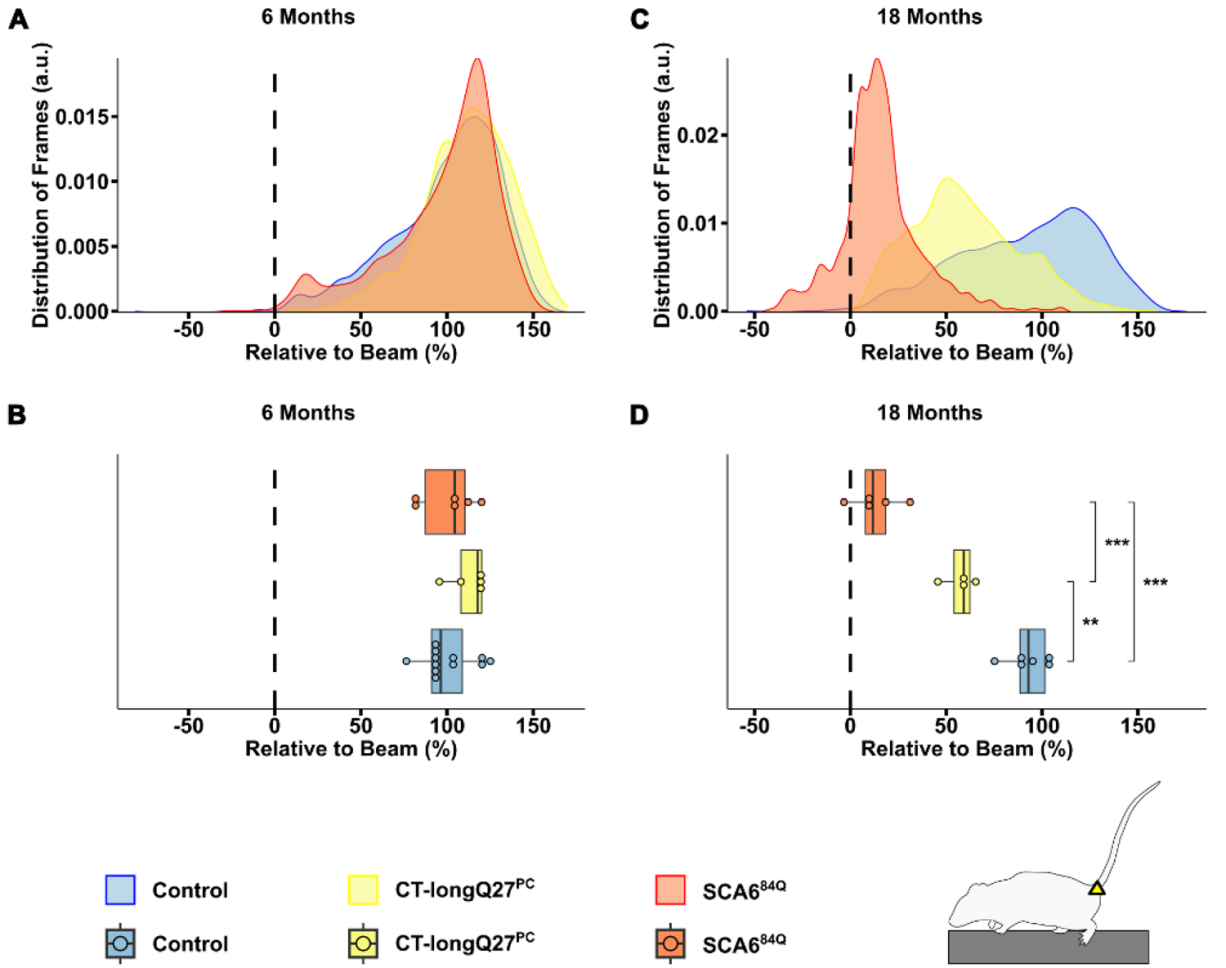
Analysis of tail base position relative to the beam by the beamwalk analysis script (BAS). The BAS utilizes the coordinates retrieved from DeepLabCut (DLC). (**A**) Density plot of tail base position (depicted in scheme as yellow triangle) relative to the top of the beam from 6 months old mice. The relative tail base position to the top of the beam in percent, where the top of the beam was represented as a vertical dashed line at 0% on the x-axis. Two different SCA6 mouse models (CT-longQ27^PC^ and SCA6^84Q^) at different disease stages (6 and 18 months), as well as control mice were compared to each other. (**B**) Boxplots of mean tail base position relative to the top of the beam from 6 months old mice. No significant difference was found between groups at 6 months old mice. (**C**) Density plot of tail base positions relative to the top of the beam from 18 months old mice. (**D**) Boxplots of mean position of the tail base relative to the top of the beam from 18 months old mice. CT-longQ27^PC^ (p = 0.0013) and SCA^84Q^ mice (p = 0.000012) were more likely to place their tail base closer to the beam compared to control mice. Additionally, SCA^84Q^ mice position their tail base lower compared to CT-longQ27^PC^ mice (p = 0.00094). Statistical significance was evaluated using two-tailed Mann–Whitney *U*-tests in (**B**) and (**D**). Bonferroni-Holm was used to correct for multiple testing. Each tested mouse is represented as individual dot. *** p < 0.001; ** p < 0.01.

**Figure 7:**
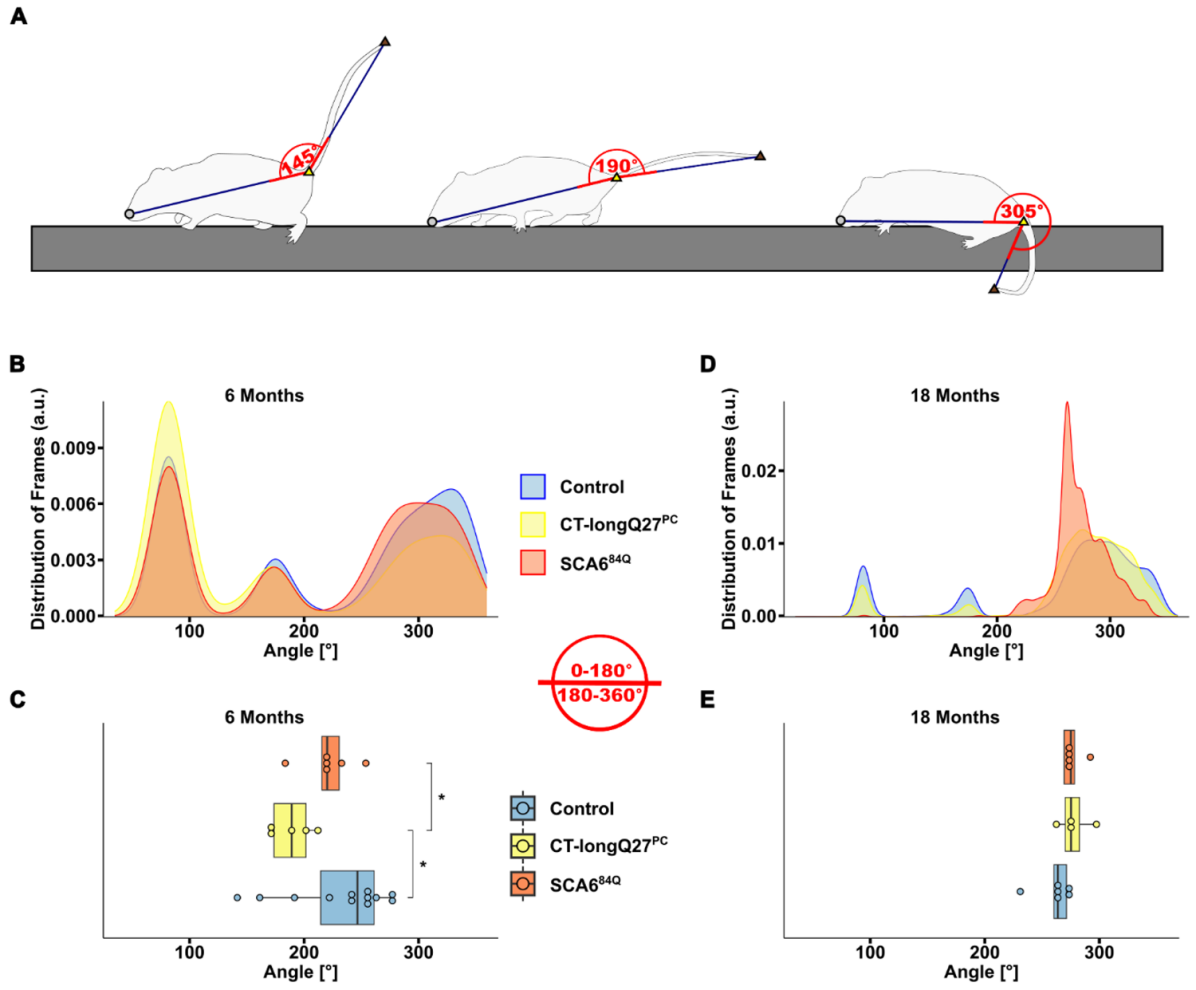
Analysis of the angle between the nose, tail base and tail tip by the beamwalk analysis script (BAS). The BAS utilizes the coordinates retrieved from DeepLabCut (DLC). (**A**) Schematic representation of the angle analyzed at the tail base, using the nose and tail tip as reference points. Angles with 145°, 190° and 305° are depicted as examples. When the tail tip (dark brown triangle) is at the same position as the nose (grey circle) with the tail base (yellow triangle) at the same height as the nose, a 0° angle forms. As the angle enlarges from 0° to 180°, indicates that the tail tip is positioned higher than the tail base as depicted in A with a tail base angle of 145º. If the tail tip is positioned below the tail base, then the angle is between 180° and 360° as depicted in (**A**) with a tail base angle of 305º. Two different SCA6 mouse models (CT-longQ27^PC^ and SCA6^84Q^) at different disease stages (6 and 18 months), as well as control mice were compared to each other. (**B**) Density plot of probability distribution of the tail base angle from 6 months old mice. (**C**) Boxplots of mean tail base angle from 6 months old mice. CT-longQ27^PC^ mice show a smaller angle at the tail base compared to both control (p = 0.029) and SCA6^84Q^ (p = 0.047) mice. (**D**) Density plot of probability distribution of the tail base angle from 18 months old mice. (**E**) Boxplots of mean tail base angle from 18 months old mice. No significant difference was found between groups at 18 months of age. Statistical significance was evaluated using two-tailed Mann–Whitney *U*-tests in (**C**) and (**E**). Bonferroni-Holm was used to correct for multiple testing. Each tested mouse is represented as individual dot. *p < 0.05.

An ataxic phenotype will often lead to a broader stride width and shorter stride length, thus resulting in an overall lower body position to compensate for the ataxia and provide more gait stability^1,23^. To investigate if the body position was closer to the beam in the SCA6 mouse models, we also measured the nose, as well as the belly, back and tail positions, namely the tail base, tail center and the tail tip, relative to the top of the beam (Fig. 1). Not surprisingly, there were no differences in nose position at 6 months of age (Fig. 4A and B), but at 18 months of age SCA^84Q^ mice (14.40 ± 1.09, p = 0.000095) were more likely to place their nose closer to the beam compared to control mice (26.96 ± 1.49, Fig. 4C and D). Comparable to the density plots for major slips, there was a clear shift to the left (i.e. nose closer to the beam) in the distribution of the nose position at 18 months of age in both SCA6 mouse models compared to controls, where the left shift was more drastic in the SCA^84Q^ mice (Fig. 4C). Similarly, no divergence in the belly (Fig. 5A and B), back (Supplementary Fig. 2A and B) or tail base (Fig. 6A and B) positions was observed in all mouse groups at 6 months of age. However, at 18 months CT-longQ27^PC^ (belly: 6.29 ± 1.85, p = 0.0021; tail base: 57.67 ± 4.91, p = 0.0013) and SCA^84Q^ (belly: − 2.24 ± 3.06, p = 0.0005; tail base: 12.27 ± 6.58, p = 0.000012) mice were more likely to place their belly and base of their tail closer to the beam compared to control mice (belly: 19.54 ± 2.31, Fig. 5D; tail base: 92.43 ± 5.07, Fig. 6D). Less obvious changes in the back position were detected except in 18 month old SCA^84Q^ mice (131.32 ± 6.73, p = 0.019), where they showed a lower position on the beam compared to control mice (155.56 ± 4.14, Supplementary Fig. 2D). There was a clear shift to the left in SCA6 belly and all 3 tail positions relative to the beam compared to controls in the density plots at 18 months of age (Figs. 5C and 6C, Supplementary Figs. 3C and 4C), indicating that SCA6 mice have a lower body position than controls. Unexpectedly, CT-longQ27^PC^ tail center (Supplementary Fig. 3B) and tip (Supplementary Fig. 4B) positions were higher above the beam than SCA^84Q^ and control mice at 6 months of age, which was confirmed by the angle at the tail base using the nose and the tail tip as reference points (Fig. 7C). In contrast, both SCA6 mouse lines demonstrated tail center (Supplementary Fig. 3D) and tip (Supplementary Fig. 4D) placement farther below the beam than controls, where SCA^84Q^ mice exhibited a graver deficit than CT-longQ27^PC^ mice. To determine if there were deviations in the tail base angle, we analyzed the angle at the tail base, utilizing the nose and tail tip as references (Fig. 7A, Supplementary Video 2). Tail base angles between 0° and 180°, indicate that the tail tip is higher than the tail base and therefore leading to a higher tail position relative to the beam. In contrast, tail base angles between 180° and 360° suggests that the tail tip is lower than the tail base and result in a tail base position below the beam. Unexpectedly, CT-longQ27^PC^ displayed slightly lower mean tail base angle suggesting a higher tail position compared to controls and SCA6^84Q^ mice at 6 months of age (Fig. 7B and C). No changes in the mean angle at the tail base using the nose and the tail tip as reference points were observed between mouse groups at 18 months of age (Fig. 7E), even though SCA^84Q^ mice demonstrated a clear peak in their probability distribution at ≈280° compared to the CT-longQ27^PC^ and control mice (Fig. 7D). Altogether these data support an ataxic phenotype in both SCA6 mouse models, where the SCA6^84Q^ line appear to manifest a more severe phenotype than the CT-longQ27^PC^ mice.

## Discussion

This study automates and enriches the data analysis of the classic beamwalk experiment using self-written scripts which we coined the beamwalk analysis script or short BAS. Together with DeepLabCut (DLC), an open source, markerless pose estimation of body parts using deep neural networks^10,11^, BAS is able to differentiate between minor and major slips and count them with ≈83% accuracy compared to manual scoring by a researcher from mice undergoing a beamwalk test (Fig. 2). Furthermore, BAS determines not only the mean relative position of any tracked body part to the top of the beam, but also the probability distribution of the marked body part position. We initially trained DLC with adult C57/Bl6 wild-type and different ataxic mice to recognize and reliably track various body parts (Fig. 1), while the mice were undergoing a beamwalk test.

Two different ataxic mouse models for spinocerebellar ataxia type 6 (SCA6) called CT-longQ27^PC^ and SCA6^84Q^, were used to verify and test the reliability and reproducibility of the DLC tracking program and BAS compared to control mice. Our self-written BAS was able to count the number of minor and major slips with an ≈83% accuracy compared to a trained researcher. Additionally, it was able to confirm an increase in the number of major slips at a post-onset disease stage (18 months) but not at pre-onset stages (6 months) in both SCA6 mouse lines as previously reported^18,21^). There was also a distinct shift from left hindpaw position from the top to below the beam in 18 month old SCA6 mice which was notably worse for the SCA6^84Q^ mice (Fig. 3E). However, less consistent changes with the severity of the disease stage and SCA6 line was found in the left forepaw position. Moreover, BAS was able to detect changes in the average and probability distribution of various body positions relative to the beam including the nose, belly, back and tail and the angle of the tail base using the nose and the tail tip as reference points to further characterize the ataxic phenotype in mice. Altogether these results verify previously reported ataxic phenotypes in both SCA6 mouse models^18,21^ tested in this study with a more detailed and unbiased analysis which allows a reliable comparative study between different mouse lines to exam differences in severity of ataxic behaviors. In this study, we were able to consistently measure an increase in the number of major left hindpaw slips and nose, belly and tail (base, center and tip) positions closer to or below the beam in SCA6 mice at 18 months of age compared to controls. Our characterization of their ataxic behaviors also revealed a more drastic motor dysfunction in SCA6^84Q^ compared to CT-longQ27^PC^ mice at 18 months of age which would not have been recognizable with the classical manual scoring method and other motor behavior tests such as the rotarod, pole test, hangwire and footprint analyses. These subtle changes in ataxic severity between the SCA6^84Q^ and CT-longQ27^PC^ mouse lines were previously not reported. The SCA6^84Q^ mouse line is a knockin mouse line with widespread expression throughout the brain with a hyperexpanded, non-physiological number of 84 polyQs in the CT of the CACNA1A gene. Whereas the CT-longQ27^PC^ line only overexpresses 27 polyQ repeats in specifically the affected cerebellar Purkinje cells, the same number of polyQs found in human patients. The number of polyQs are known in patients to be directly correlated to the severity of the disease and inversely to the age of onset. The widespread expression and high number of polyQs incorporated in the SCA6^84Q^ mice may mechanistically contribute to the degeneration of neurons, buildup of aggregates and thus leading to a slightly more severe ataxic phenotype compared to the CT-longQ27^PC^ line.

BAS was developed to count paw slips comparable to a human rater, as well as analyze detailed gait parameters not typically investigated in the beamwalk test. It therefore sets itself apart from what one may obtain from performing the analysis with programs that target only gait parameters such as Visual Gait Lab^24^, LocoMouse^7^ and DigiGait^4^. As these were developed to process videos of animals freely walking in arenas or on a treadmill where paw slips would not occur, it is highly unlikely that they could provide a proper analysis of paw slips. Moreover, like many other gait analysis tools, Visual Gait Lab is made to handle videos taken from below rather than the side. Filming the beamwalk test from below would be highly unusual and likely difficult to work with as one would not see much of the animal.

Others have approached detailed beamwalk analysis by using behavioral classifiers such as SimBA^25^ and JAABA^16^, or by making general visualizations of the tracked position of individual body parts. Bidgood et al.^26^, used SimBA to classify periods of walking and falls in a model of Parkinson’s disease. This would not have been sufficient for the analysis of the current study, as the analysis was specifically focused on videos where animals crossed the beam without longer breaks and without falling off the beam. The aim of the current study was in part to investigate presence of discreet motor impairments in younger animals, where breaks in walking and falls were rare to the point of the latter representing outlier cases. Wahl et al.^16^ used a similar approach to classify stops and falls, using JAABA, but also included slips in their analysis. Notably, BAS goes one step further and was developed to provide additional details regarding not only the presence of a slip but also their relative severity. Finally, the approach of Lang et al.^12^, provided a visualization of the position of various body parts, and ultimately quantified a movement rhythmicity measurement. While interesting, the current models prominent hind paw impairments have so far made such measurements difficult to implement in a satisfactory way. As such, compared to other published beamwalk analysis approaches, BAS provides a satisfactory automated replacement of the classical slip score, while providing detailed information on both slips and gait parameters in a way others have not achieved.

In this study, we showed that the combination of DLC^10,11^ and BAS provides researchers with an unbiased, consistent and efficient method to analyze ataxic abnormalities in mice. It also allows a more detailed characterization of various body positions relative to the beam to provide more subtle evidence for ataxia onset during the early stages of the disease or in mild cases of ataxia. Finally, our BAS depicts the probability distribution of different body parts relative to the beam and determines the probability that a particular body part is below or closer to the beam. This visual shift in the distribution of the density plots allows one to gain a deeper insight into the data, where the effect or trend in redistribution was lost in the average position relative to the beam. In summary, the use of machine learning and neural networks such as DLC with BAS provides researchers with an objective, cost and time efficient and reliable system to compare and characterize mouse models with motor deficits using the classical beamwalk experiment. More importantly, this system can be applied to compare the effectiveness of various pharmacological, genetic or stimulation (i.e. optogenetic or deep brain stimulations) treatments on motor dysfunction.

## Methods

### Mouse lines

All mice were group housed with two to five mice inside conventional type III cages in the behavior laboratory on a 12/12 h normal light/dark cycle with food and water available ad libitum for the duration of the testing period. A paper tube and nesting paper were also present in each cage for enrichment. Cleaning of the cages was executed at least 2 days prior to testing and not during the testing period to avoid stress and distraction. All tests were performed during their dark phase between 6 pm and 4 am under artificial white light to minimize disruption of the sleep cycle. Mice were acclimated for at least 7 days to the behavior laboratory before testing. SCA6^84Q^ mice were a gift from Dr. Alanna Watt (McGill University, Montreal, Canada) with prior consent from Dr. Huda Zoghbi (Baylor College of Medicine, Houston, Texas).

Presence of transgene expression was detected by polymerase chain reaction analysis CT-short^PC^ and CT-longQ27^PC^ as followed^18,27,28^. Forward 5 CGACCACTACCAGCAGAACA 3ˊ, reverse, 5ˊ CCACGGACTGAGAGTTAGGC 3ˊ and Tg^Cre^ forward, 5ˊ ATTCTCCCACCACCGTCAGTACG 3ˊ, reverse, 5ˊ AAAATTTGCCTGCATTACCG 3ˊ. SCA6^84Q^ transgene presence was detected using forward 5ˊ ACGTGTCCTATTCCCCTGTGATCC 3ˊ and 5ˊ TGC-ACCCCGCGGCCGCTTGTGTC 3ˊ and reverse 5ˊ ACCAGTCGTCCTCGCTCTC 3ˊ.

### Beamwalk

The age, sex and number of mice used in each mouse line is described in Table 1. Mice were trained to walk across a 1 × 2  ×  63 cm (width  ×  height  ×  length) beam positioned 60 cm above the ground towards a dark enclosure at one end. Mice were habituated and trained on the beamwalk on the test day. Two markings on the beam, spaced 39 cm apart indicated the trial distance. Mice were placed on a starting platform outside the first marking and were allowed to cross the beam at their own initiative. Videos were recorded on a Sony HDR-CX730E camera with 50 frames per second, an ISO of 3500 and in 1920 by 1080 pixels with a slight increase in white balance. The camera was placed around 65 cm away from the beam. To illuminate the beam from both sides, 2 floodlights were positioned 80 cm away from the beam. On average, 3 trials were recorded and only successful trials where mice reached the end marking without falling or turning back were included into the analysis.

**Table 1:** Description of mice used in beamwalk test.

	Age group	Age (months), Mean ± SEM	Male, female	N_Mice_	n_trials_	n_frames_
Control	6	5.9 ± 0.09	9, 3	12	41	28,414
	18	17.8 ± 0.19	3, 3	6	20	19,547
CT-longQ27^PC^	6	6.6 ± 0.01	4, 2	6	18	13,864
	18	19.2 ± 0.06	2, 2	4	9	19,042
SCA6^84Q^	6	5.9 ± 0.00	3, 3	6	20	16,359
	18	19.3 ± 0.36	4, 1	5	7	9193

### Pose estimation using DeepLabCut

In this study DeepLabCut (DLC) (version 2.2.1) was used for training a neural network capable of tracking multiple body parts and positions along the beam^10,11^. Specifically, 1105 frames were taken from 45 videos evenly selected from different age, sex and genotype groups to cover animals with differing motoric abilities. These frames were labeled and then 95% of them were used for training. Exclusively for training the network, lighting conditions, frames per second, frame size, number of frames and their selection method, as well as the set-up recorded varied between videos. A ResNet-50-based neural network^29^ with default parameters for 650,000 number of training iterations was used. After training, DLCs network evaluation indicated that the network had a test error of 2.77 pixels and a train error of 6.11 pixels (as validated with 6 number of shuffles and an image size of 1920 by 1080 pixels, without using any p-cutoff value). The network was trained to track four positions along the beam as well as the positions of the animal’s left eye, nose, paws, belly, back and three points along the tail (Fig. 1). Using a custom written R script through DLC obtained filtered .csv-files, different p-cutoff values were used for the tracked labels to condition the x- and y-coordinates for further analysis, as well as the change in position over time. Conditioning sufficed with a p-cutoff value of around 0.2. Tracking of the videos for the SCA6 mice were performed blindly.

### Analysis using R

For the analysis of the obtained tracking data, the open-source software R (version 4.2.2) in combination with RStudio (version 2023.03.0 + 386) were used, as well as the following packages: photobiology (version 0.10.17), ggplot2 (version 3.4.2), ggpubr (version 0.6.0) and svglite (version 2.1.1).

The detailed functions of the script are described through code annotations in the supplied files at GitHub (https://github.com/RUB-Behavioral-Neuroscience/Beamwalk_Analysis_Script). A more general description is given here. The entire analysis is based on the absolute position, meaning the x- and y-coordinates, of the tracked labels (Fig. 1) obtained by DeepLabCut (DLC) filtered .csv-files that were conditioned to exclude inaccurate tracking using a custom written R script. For the analysis, only frames from the coordinates of the analyzed label that were between the start (light green square) and stop (dark green square) markings were considered. Furthermore, stationary frames were excluded from the analysis. An animal was considered stationary when the belly (blue circle), back (green circle) and tail base (yellow triangle) each did not change their position more than 1.5 pixels compared to the previous frame. The top of the beam was calculated as a hypothetical straight line through the coordinates that tracked the top of the beam on the left (dark blue square) and right side (light blue square).

Specific focus was put on the tracked positions of the left forepaw (pink star, Supplementary Fig. 1) and hindpaw (orange star, Fig. 3), to construct an automated slip classification. For this, frames that were below the calculated top of the beam were considered. Different thresholds were used for the minor and major slip classification for the left hind- and forepaw. For the hindpaw, tracked points needed to be between 20 to 50% below the beam to be considered as a minor slip and below 50% to be considered as a major slip. For the forepaw, tracked points needed to be between 10 and 30% below the beam to be considered as a minor slip and below 30% to be considered as a major slip. These thresholds were determined empirically through repeated testing.

From there on, peak and valley detection functions from the photobiology library were used to detect peaks and valleys in the data. In brief, the script considered the presence of peaks using different criteria, such as the width and spacing, as well as the number of consecutive frames below a given threshold, to assess whether one or multiple slips occurred during the period that body part was below that threshold. This process was similar, but not identical, for both minor and major slip classification.

Besides classifying slips, various body parts were tracked and depicted as a percentage relative to the beam. For example, 0% was at the top of the beam and 100% was equivalent to one beam height away from the top of the beam. In addition, the angle at the tail base relative to the nose (grey circle) and tail tip (dark brown triangle) was used as a rudimentary posture analysis. If the tail tip was for example occluded, the tail center (light brown triangle) was used to determine the angle as a back-up. The body area of an animal was also determined in each trial by calculating an irregular rectangle between the nose, belly, tail base and back, and then averaged for each animal. Other parameters included the trial length, which was any frame of a given body part between the start and stop markings, as well as the time spend below the beam from that trial length.

Variances were not equal between the groups being compared, and therefore nonparametric tests were performed. Furthermore, normality was not assumed, hence non-parametric tests were performed. Statistical significance was evaluated using two-tailed Mann–Whitney *U* tests. Bonferroni-Holm was used to correct for multiple testing. Statistical significance was indicated with ***p < 0.001, **p < 0.01 and *p < 0.05 and is reported in all figure legends. All values are reported as mean ± standard error of mean with p-values in Supplementary Table 3.

### Ethics declaration

The present study was carried out in accordance with the European Communities Council Directive of 2010 (2010/63/EU) for the care of laboratory animals and approved by a local ethics committee (Bezirksamt Arnsberg) and the animal care committee of North Rhine-Westphalia, Germany, based at the LANUV (Landesamt für Umweltschutz, Naturschutz und Verbraucherschutz, Nordrhein-Westfalen, D-45659 Recklinghausen, Germany). The study was supervised by the animal welfare commission of the Ruhr-University Bochum. All efforts were made to minimize the number of mice used for this study by applying G*power 3.1 analyses (ANOVA: 0.05 alpha error probability, 0.95 1-beta error probability). This study is reported in accordance to the ARRIVE guidelines.

### Supplementary Information

Supplementary Video 1.

Supplementary Video 2.

Supplementary Information.

## Data Availability

All data will be made available by Melanie D. Mark (melanie.mark@rub.de) upon reasonable request. Scripts, images, tables and used software are available at GitHub (https://github.com/RUB-Behavioral-Neuroscience/Beamwalk_Analysis_Script).
