# Supplementary material for: Phenotypic analysis of ataxia in spinocerebellar ataxia type 6 mice using DeepLabCut
Source: Sci Rep. 2024 Apr 13;14:8571. doi: 10.1038/s41598-024-59187-0 (PMC11014858; doi:10.1038/s41598-024-59187-0)
Supplement: Supplementary file 3 — Supplementary Information. [file 41598_2024_59187_MOESM3_ESM.docx]

**Supplementary Information**

Supplementary video 1. An example of video of a control mouse performing the beamwalk test and demonstrating a minor and major slip with their left hindpaw.

Supplementary video 2. An example video depicting the angle at the tail base, utilizing the nose and tail tip as references. 90°, 180° and 270° are shown as examples.

**Supplementary Table 1.** Mean body size of mice.


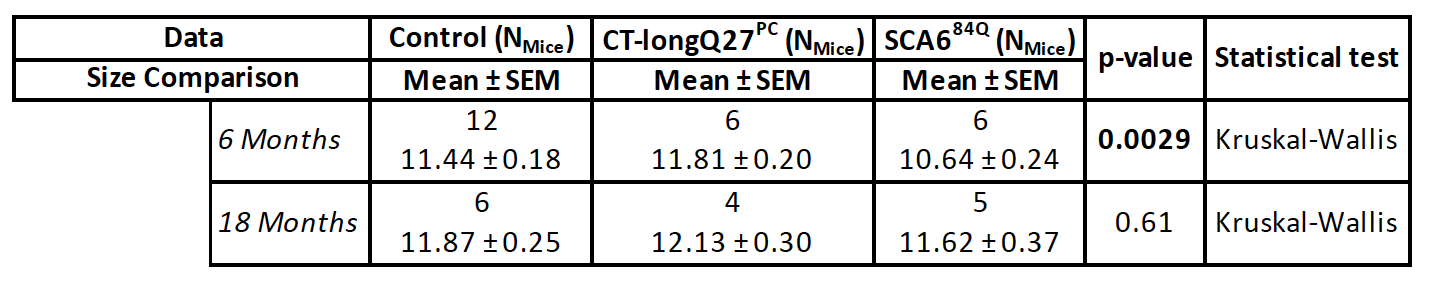


Statistically significant p-values are indicated in bold.

**Supplementary Table 2.** Comparison of minor and major slips counted by BAS versus Researcher.
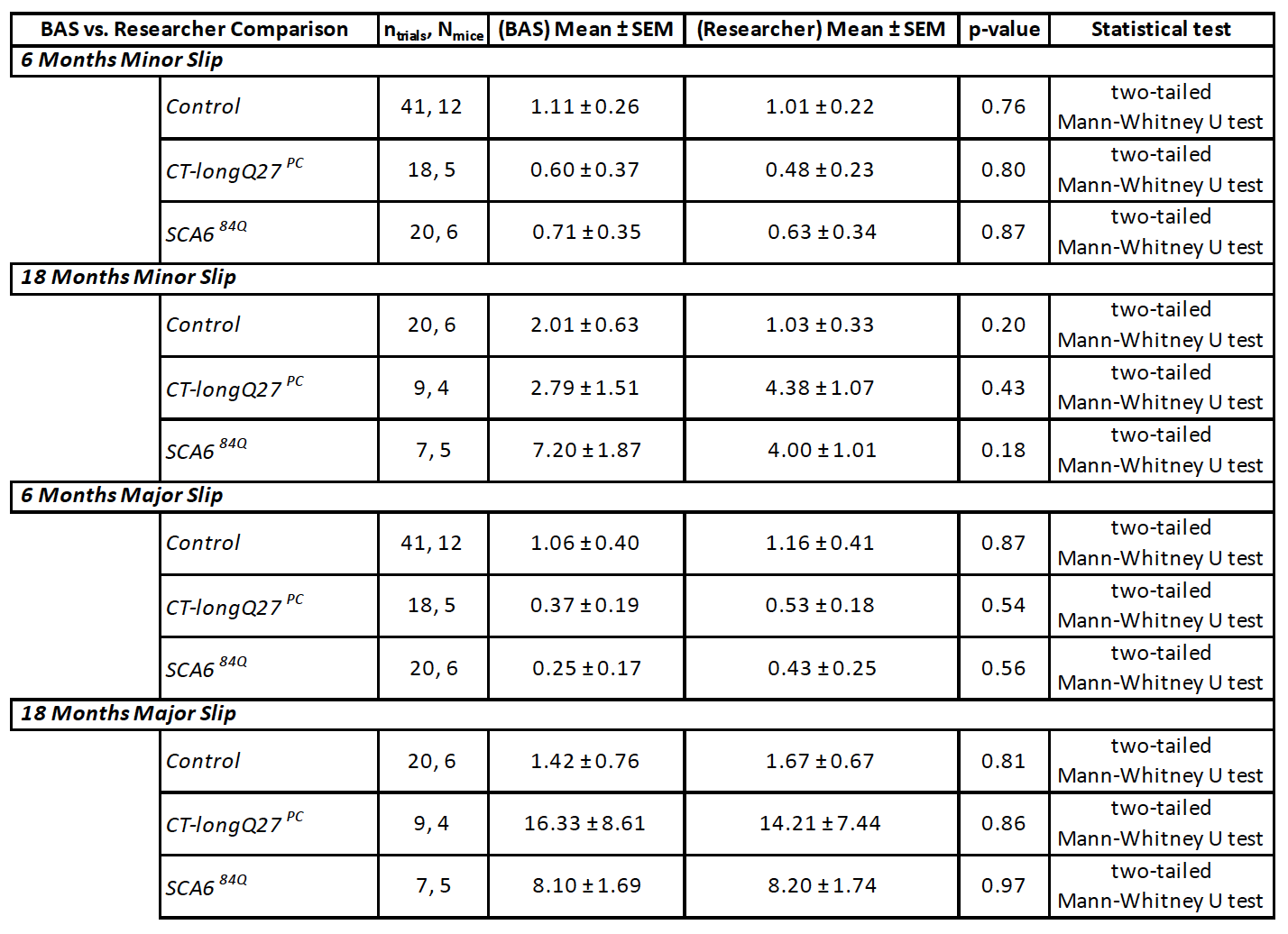


**Supplementary Table 3.** Mean position of different body parts at 6 and 18 months old control, CT-longQ27^PC^ and SCA6^84Q^ mice.


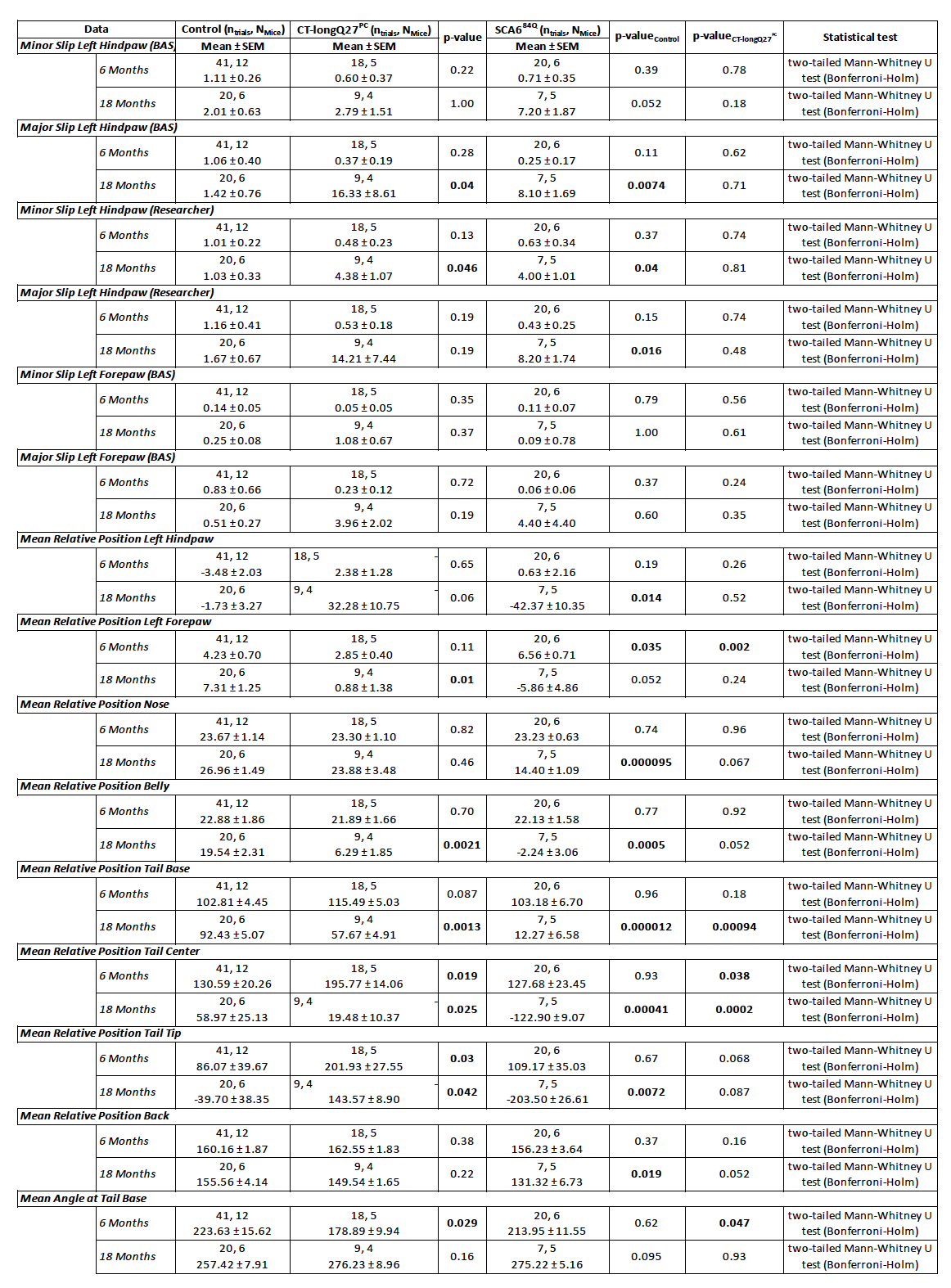


Statistically significant p-values are indicated in bold.


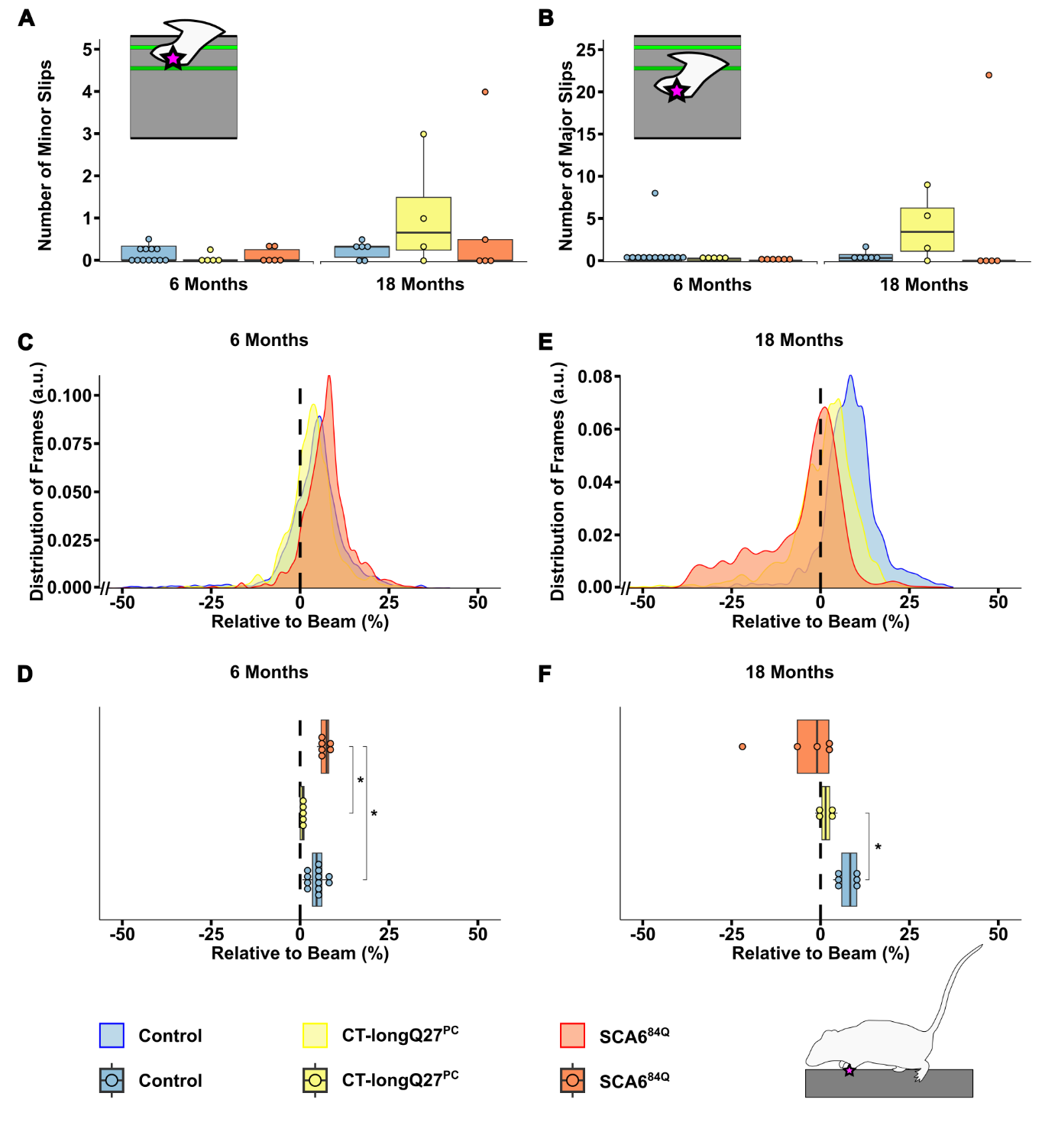


**Supplementary Figure 1:** **Classical and advanced analysis of the left forepaw by the beamwalk analysis script (BAS).** The BAS utilizes the coordinates retrieved from DeepLabCut (DLC) to classify vertical left forepaw (depicted in scheme as pink star) displacements as minor (A) and major (B) slips. For this analysis, minor slips were defined as paw positions between 10% (upper green line) and 30% (lower dark green line) below beam. Any slips > 30% below the top of the beam were defined as major slips. The left forepaw position relative to the top of the beam in percent was analyzed in C, D, E and F, where a vertical dashed line represents 0% (the top of the beam) on the x‑axis. Two different SCA6 mouse models (CT‑longQ27^PC^ and SCA6^84Q^) at different disease stages (6 and 18 months), as well as control mice were compared to each other. **A** Boxplots of the mean number of minor slips over all trials. No significant difference was found between groups at 6 months of age. **B** Boxplots of the mean number of major slips over all trials at 18 months of age. No significant difference was found between groups at 18 months of age. **C** Density plot displaying the probability distribution of the left forepaw position relative to the top of the beam from 6 months old mice. **D** Boxplots of the mean left forepaw position relative to the top of the beam from 6 months old mice. SCA6^84Q^ mice placed their left forepaw relatively higher on the beam compared to CT‑longQ27^PC^ (p = 0.002) and control (p = 0.035) mice. **E** Density plot displaying the probability distribution of the left forepaw position relative to the top of the beam from 18 months old mice. **F** Boxplots the mean left forepaw position relative to the top of the beam from 18 months old mice. The left forepaw of CT‑longQ27^PC^ mice were placed lower on the beam compared to control mice (p = 0.0072). Statistical significance was evaluated using two‑tailed Mann‑Whitney U‑tests in A, B, D and F. Bonferroni‑Holm was used to correct for multiple testing. Each tested mouse is represented as individual dot. * p < 0.05


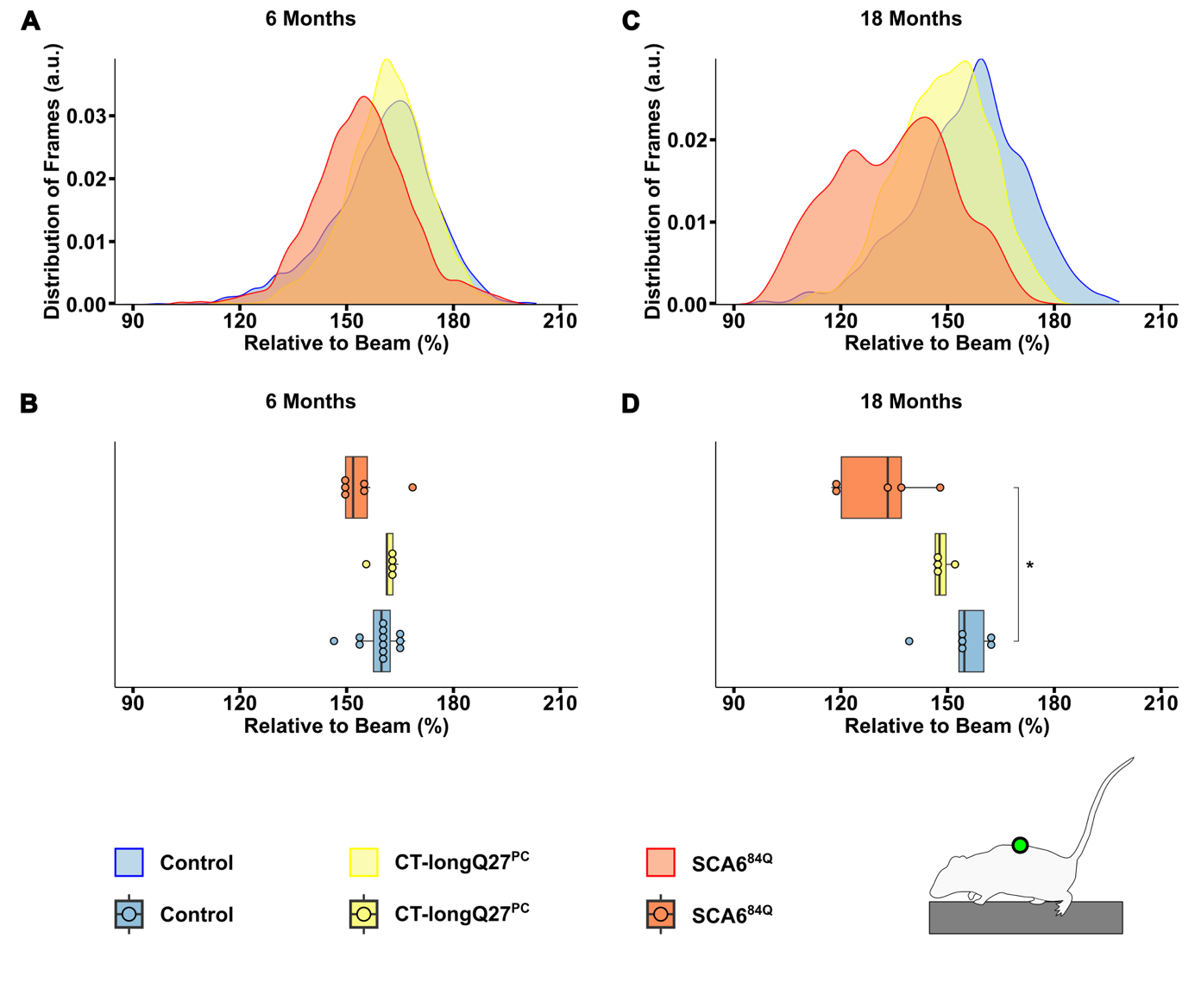


**Supplementary Figure 2:** **Analysis of back position relative to the beam by the beamwalk analysis script (BAS).** The BAS utilizes the coordinates retrieved from DeepLabCut (DLC). **A** Density plot of back position (depicted in scheme as green circle) relative to the top of the beam from 6 months old mice. The relative back position to the top of the beam in percent, where the top of the beam is represented as a vertical dashed line at 0% on the x‑axis. Two different SCA6 mouse models (CT‑longQ27^PC^ and SCA6^84Q^) at different disease stages (6 and 18 months), as well as control mice were compared to each other. **B** Boxplots of mean relative back position to the top of the beam from 6 months old mice. No significant difference was found between groups at 6 months of age. **C** Density plot of back position relative to the top of the beam from 18 months old mice. **D** Boxplots of mean relative belly position to the top of the beam from 18 months old mice. SCA^84Q^ mice (p = 0.019) were more likely to place their back closer to the beam compared to controls. Statistical significance was evaluated using two‑tailed Mann‑Whitney U‑tests in B and D. Bonferroni‑Holm was used to correct for multiple testing. Each tested mouse is represented as individual dot. * p < 0.05


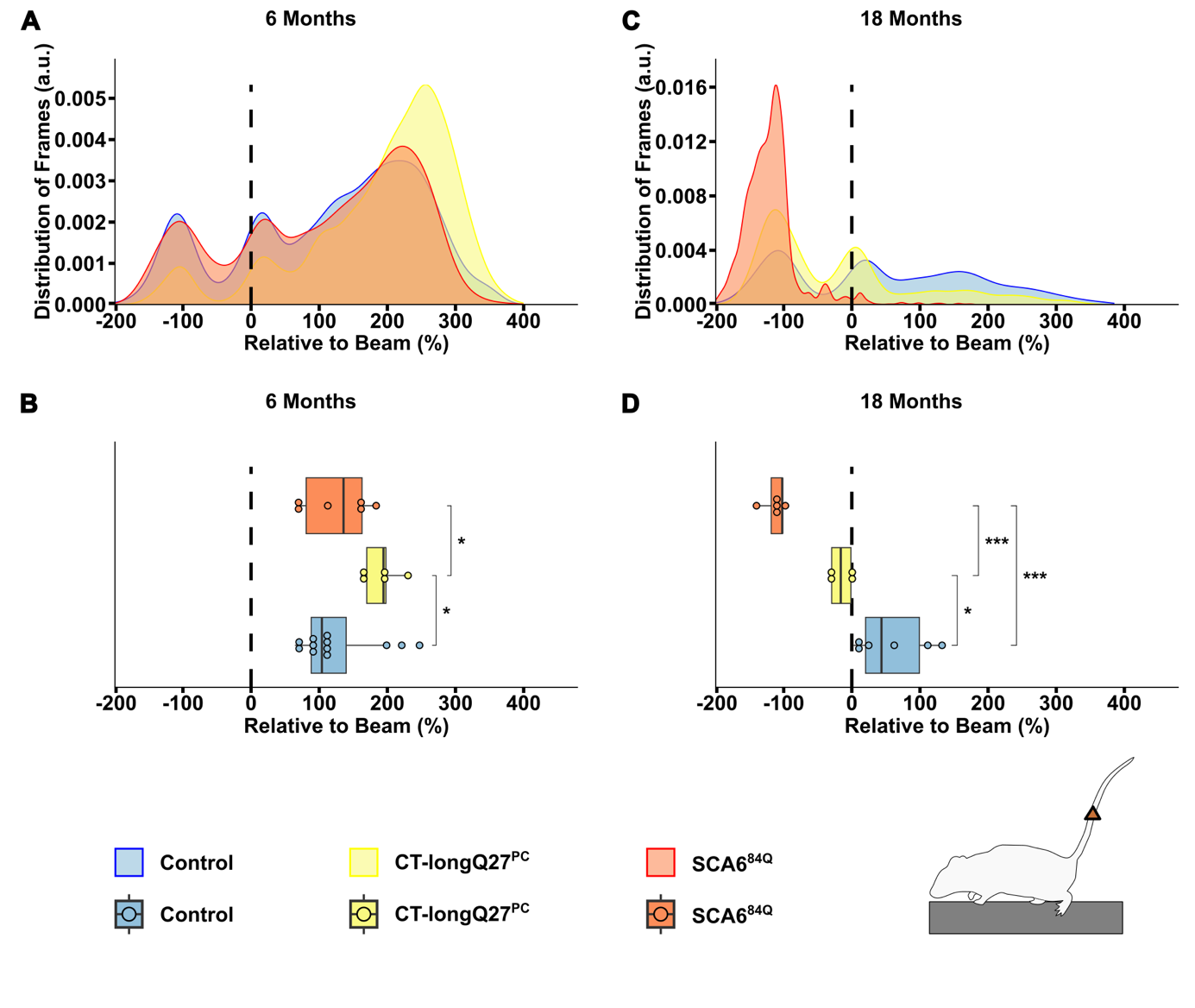


**Supplementary Figure 3:** **Analysis of tail center position relative to the beam by the beamwalk analysis script (BAS).** The BAS utilizes the coordinates retrieved from DeepLabCut (DLC). **A** Density plot of tail center position (depicted in scheme as light brown triangle) relative to the top of the beam from 6 months old mice. The relative tail center position to the top of the beam in percent, where the top edge of the beam was represented as a vertical dashed line at 0% on the x‑axis. Two different SCA6 mouse models (CT‑longQ27^PC^ and SCA6^84Q^) at different disease stages (6 and 18 months), as well as control mice were compared to each other. **B** Boxplots of mean tail center position relative to the top of the beam from 6 months old mice. CT‑longQ27^PC^ held their tails higher than SCA6^84Q^ (p = 0.038) and control (p = 0.019) mice at 6 months old. **C** Density plot of tail center positions relative to the top of the beam from 18 months old mice. **D** Boxplots of mean position of the tail center relative to the top of the beam from 18 months old mice. CT‑longQ27^PC^ (p = 0.025) and SCA^84Q^ mice (p = 0.00041) were more likely to place their tail center closer or below the beam compared to control mice. Additionally, SCA^84Q^ mice positioned their tail center lower compared to CT‑longQ27^PC^ mice (p = 0.0002). Statistical significance was evaluated using two‑tailed Mann‑Whitney U‑tests in B and D. Bonferroni‑Holm was used to correct for multiple testing. Each tested mouse is represented as individual dot. *** p < 0.001; * p < 0.05


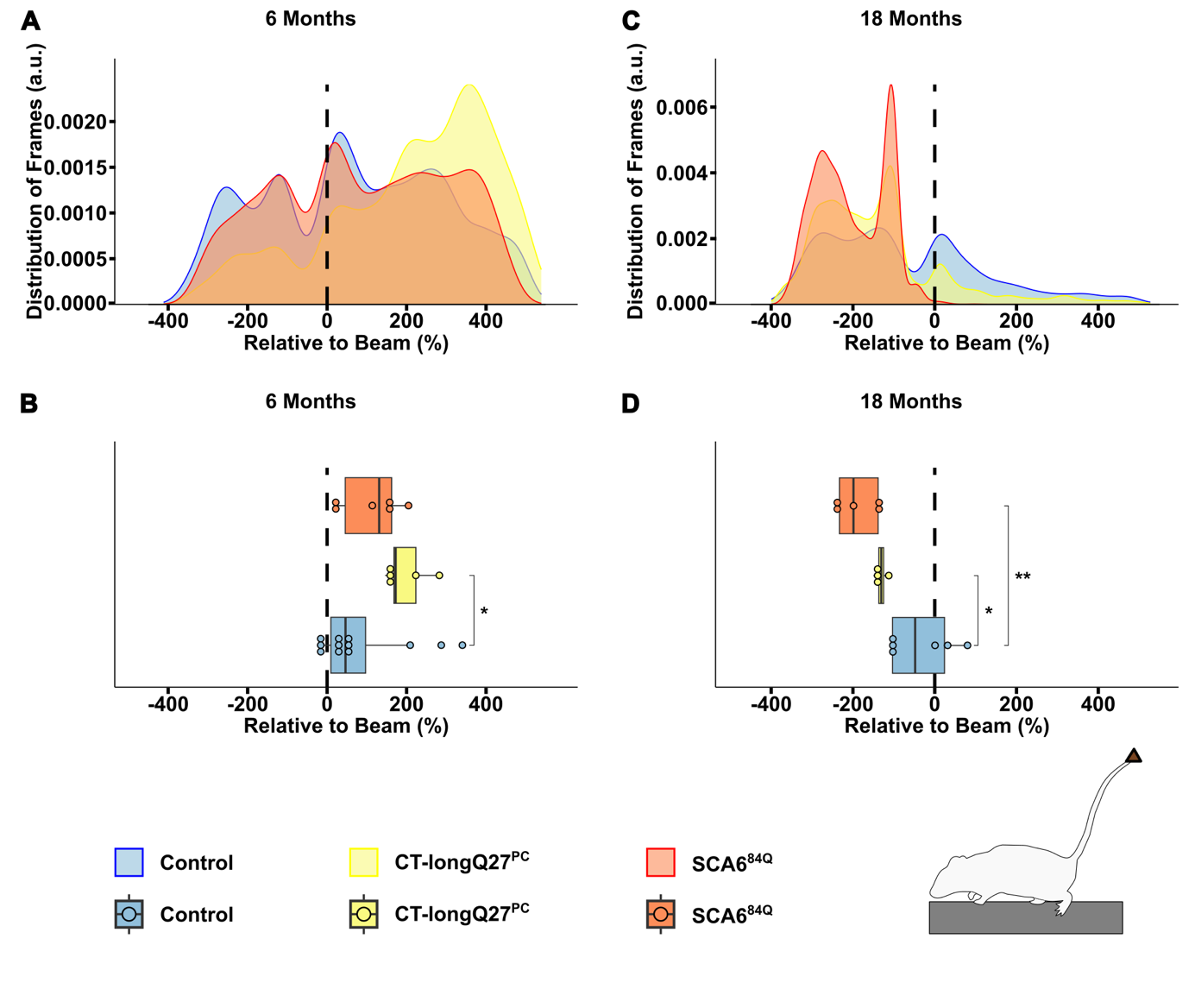


**Supplementary Figure 4:** **Analysis of tail tip position relative to the beam by the beamwalk analysis script (BAS).** The BAS utilizes the coordinates retrieved from DeepLabCut (DLC). **A** Density plot of tail tip position (depicted in scheme as brown triangle) relative to the top of the beam from 6 months old mice. The relative tail tip position to the top of the beam in percent, where the top edge of the beam was represented as a vertical dashed line at 0% on the x‑axis. Two different SCA6 mouse models (CT‑longQ27^PC^ and SCA6^84Q^) at different disease stages (6 and 18 months), as well as control mice were compared to each other. **B** Boxplots of mean tail tip position relative to the top of the beam from 6 months old mice. CT‑longQ27^PC^ held their tails higher than control (p = 0.03) mice at 6 months old. **C** Density plot of tail tip positions relative to the top of the beam from 18 months old mice. **D** Boxplots of mean position of the tail tip relative to the top of the beam from 18 months old mice. CT‑longQ27^PC^ (p = 0.042) and SCA^84Q^ mice (p = 0.0072) were more likely to place their tail tip closer or below the beam compared to control mice. Statistical significance was evaluated using two‑tailed Mann‑Whitney U‑tests in B and D. Bonferroni‑Holm was used to correct for multiple testing. Each tested mouse is represented as individual dot. ** p < 0.01; * p < 0.05
